# Silicon supplied via foliar application and root to attenuate potassium deficiency in common bean plants

**DOI:** 10.1038/s41598-021-99194-z

**Published:** 2021-10-04

**Authors:** Marcilene Machado dos Santos Sarah, Renato de Mello Prado, Jonas Pereira de Souza Júnior, Gelza Carliane Marques Teixeira, João Carlos dos Santos Duarte, Robson Luis Silva de Medeiros

**Affiliations:** School of Agricultural and Veterinarian Sciences, São Paulo State University (Unesp), Via de Acesso Prof. Paulo Donato Castellane s/n, Jaboticabal, 14884-900 Brazil

**Keywords:** Abiotic, Plant immunity, Plant physiology, Plant stress responses

## Abstract

Potassium (K) deficiency affects physiological performance and decreases vegetative growth in common bean plants. Although silicon (Si) supplied via nutrient solution or foliar application may alleviate nutritional stress, research on the bean crop is incipient. Thus, two experiments were carried out: initially, a test was performed to determine the best source and foliar concentration of silicon. Subsequently, the chosen Si source was supplied in nutrient solution via roots or foliar application to verify whether Si supply forms are efficient in alleviating the effects of K deficiency. For these purposes, a completely randomized 2 × 3 factorial design was used, with two levels of K: deficient (0.2 mmol L^−1^ of K) and sufficient (6 mmol L^−1^ of K); and Si: in nutrient solution via roots (2 mmol L^−1^ of Si) or foliar application (5.4 mmol L^−1^ of Si) and control (0 mmol L^−1^ of Si). Our findings revealed that Si supplied via foliar spraying using the source of sodium silicate and stabilized potassium at a concentration of 5.4 mmol L^−1^ was agronomically viable for the cultivation of bean plant. K deficiency, when not supplied with silicon, compromised plant growth. Moreover, root-and-foliar-applied Si attenuated the effects of K deficiency as it increased chlorophylls and carotenoids content, photosynthetic activity, water use efficiency and vegetative growth. For the first time, the role of Si to mitigate K deficiency in the bean crop was evidenced, with a view to further research on plants that do not accumulate this beneficial element.

## Introduction

Potassium activates more than 60 enzymes and directly participates in protein synthesis in vegetables^1,2^. This nutrient acts in carbohydrate translocation from the shoot to the roots and stimulates osmotic regulation by reducing water loss through transpiration, balancing the opening and closing of stomata, and increasing leaf turgor and cell expansion^1–3^.

Potassium deficiency in bean plants (*Phaseolus vulgaris* L.) is common worldwide, inducing chlorosis on the edges of older leaves, which progresses to necrosis^1,4^. At this stage, reactive oxygen species that degrade chlorophyll increase, thus decreasing photosynthesis and increasing transpiration due to the inefficient water use^1,5,6^. This stress reduces crop development, pod production per plant^1^, root volume, stem growth, leaf area and number of leaves^4^.

Studies show that Si can mitigate K deficiency stress, because it increases the levels of chlorophyll and antioxidant compounds (carotenoids). This favours photosynthesis rates^5–9^ and decreases transpiration, thereby increasing the efficiency of water use^6,10^ and dry matter production^6,8^. One strategy to alleviate nutritional K deficiency is Si supply, although its interaction with K has been less investigated than that with N and P^11^.Studies show that Si supply can alleviate K-deficiency stress in puddles because it stimulates K uptake and accumulation^8,9,12^, possibly increasing the expression of K-transporting genes (OsHAK5, OsAKT1 and OsSKOR)^13^. Furthermore, Si decreases cell oxidation^8^ and increases the contents of chlorophyll and non-enzymatic antioxidant compounds such as carotenoids^5,9,14^. These benefits of Si increase photosynthetic rates^5–9^, decreasing transpiration and increasing water use efficiency^6,10^ and consequently dry matter production of K-deficient plants^6,8,9^. However, information about the relationship between Si and K deficiency stress in bean plants is non-existent. A number of studies on other species indicate that supplying Si nutrient solution alleviates K deficiency in soybean^8^, sorghum^5,6^, barley crops^14^ and maize plants^9^, but there are no reports on Si foliar spray and this nutritional disorder in any legume species. In fact, doubts remain as to the best source and concentration of Si for foliar spray in bean plants. The optimal foliar uptake of Si depends on the source and concentration, as it affects the polymerization rate of this element in solution and, consequently, the crop response^15,16^, especially in non-Si-accumulating species such as legumes.

Therefore, a number of questions must be answered. First, it is whether Si foliar spray on bean plants is agronomically feasible depending on the source and concentration of the element. The hypothesis is that Si supply alleviates K deficiency due to increased chlorophyll content, photosynthesis and water use efficiency of the bean plant. In this case, mitigating K deficiency is more evident with Si supply via nutrient solution (roots) compared to foliar applications, although foliar spray can also reduce plant deficiency.

This study determines the best source and concentration of Si for foliar application and investigates whether supplying this source via nutrient solution is efficient to alleviate the K-deficiency stress in bean plants. If this hypothesis is supported, it will elucidate how Si works to overcome K deficiency, which is common in bean crops, with additional practical implications on how best to apply this element in the crop.

## Results

### Foliar application of Si and its effect on bean plants

**Figure 1:**
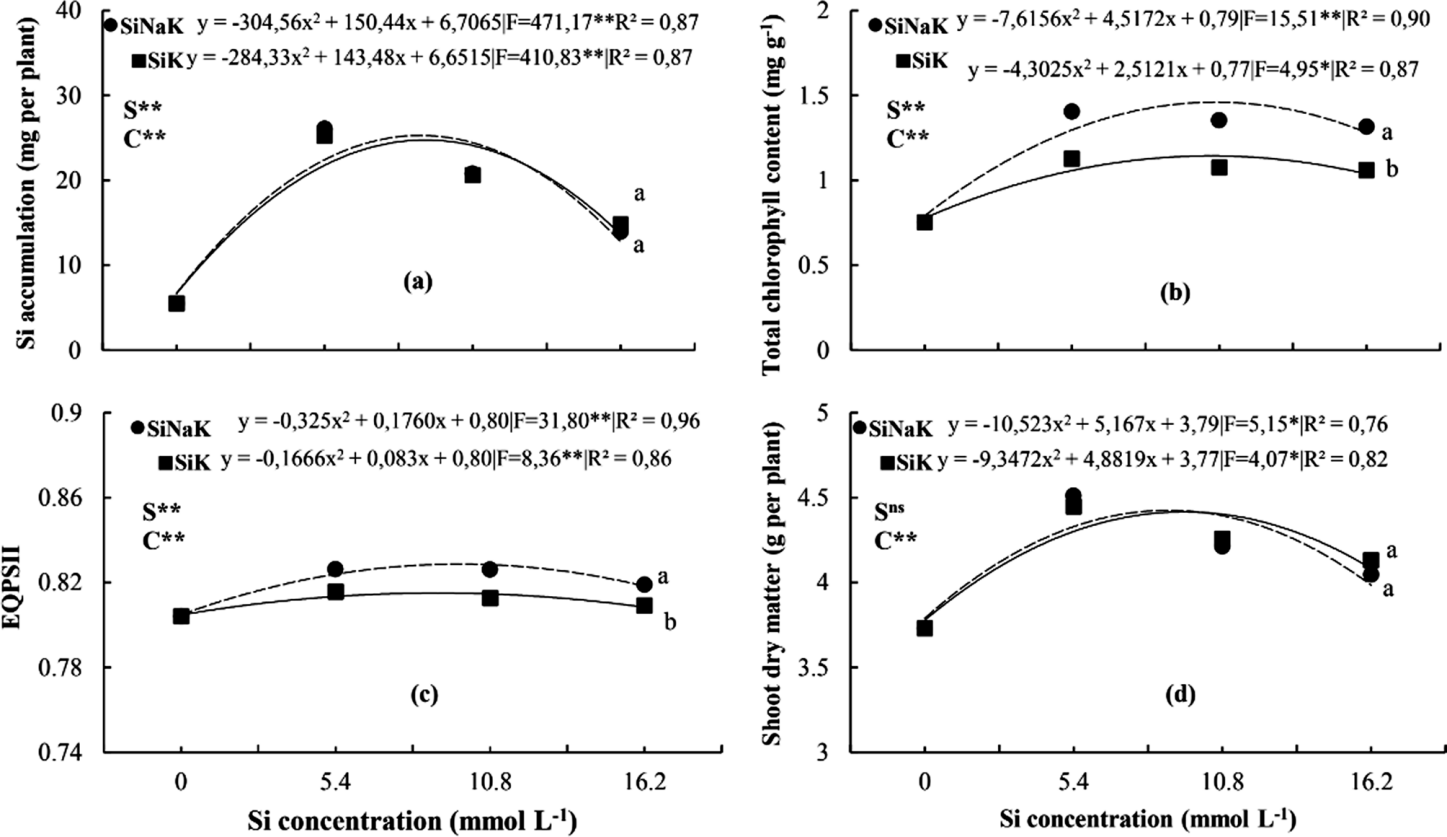
Si accumulation (shoot) (**a**), total chlorophyll content (**b**), QEPII—Quantum efficiency of photosystem II (**c**) and shoot dry matter (**d**) of bean plants cultivated with different sources (S) of Si [sodium and potassium silicate stabilized (SiNaK) and potassium silicate without stabilizer (SiK)] and increasing concentrations (C) of leaf-applied Si.**Significant at 1% probability; ^ns^no significant at 5% probability; Different letters in the same Si concentration indicate differences (P < 0.05, Tukey test) between treatments.

The increase in the concentration of Si applied to the leaves increased the element accumulation, total chlorophyll content, quantum efficiency of photosystem II and shoot dry matter of bean plants, regardless of the source used (Fig. 1a–d).

The Si concentrations applied to the leaves as SiK and SiNaK sources, which resulted in the maximum Si accumulation, total chlorophyll content, quantum efficiency of photosystem II and dry matter were: 8.93 and 8.57; 10.36 and 10.36; 8.57 and 9.64; and 9.29 and 8.57 mmol L^−1^ of Si, respectively (Fig. 1). Based on the results, SiNaK and SiK performed equally in increasing Si in the plant; however, SiNaK produced better results in total chlorophyll content and quantum efficiency of photosystem II, albeit not enough to affect dry matter (Fig. 1). The use of SiNaK source at a concentration equal to 5.4 mmol L^−1^ of Si was associated with 90% of maximum dry matter production, being a viable option for foliar spray of the beneficial element in bean plants.

### Potassium and silicon

**Figure 2:**
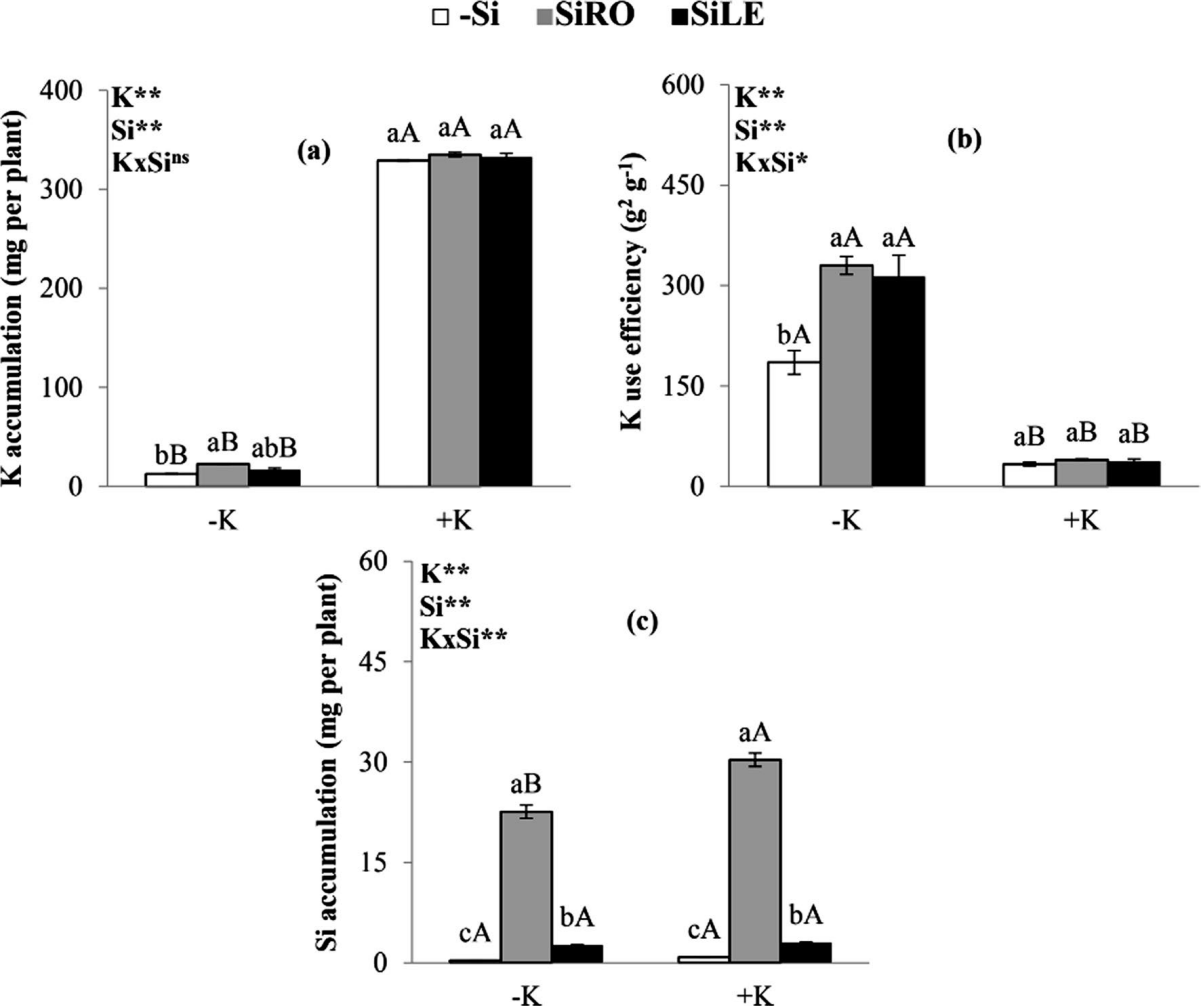
K accumulation (shoot + root) (**a**), K use efficiency (**b**) and Si accumulation (shoot + root) (**c**) of bean plants cultivated in a hydroponic system under deficiency (−K) and sufficiency (+K) of K, with Si supplied via nutrient solution (root) (SiRO), leaf spraying (SiLE), and control (–Si). The error bars in the figures represent standard error. Different letters, lower case between the supply of Si in the same concentration of K, and upper case between concentrations of K in the same form of supply of Si, indicate differences (P < 0.05, Tukey's test) between treatments.

To cultivate bean plants in a K-deficient nutrient solution resulted in less nutrient accumulation, regardless of Si treatments and control (–Si) (Fig. 2a). Only the application of silicon in the form of SiRO in bean plants cultivated under K deficiency in relation to its sufficiency increased the accumulation of potassium (Fig. 2a). In K-deficient plants, the treatment using SiRO and SiLE increased the macronutrient efficiency compared to that in the controls (Fig. 2b). This indicates the beneficial effect of Si in improving K uptake and Si use efficiency, contributing to alleviating nutritional stress in the bean plant.

Potassium-deficient beans plants when compared to K-sufficient plants had decreased Si accumulation only when Si was supplied by roots (Fig. 2c). The use of Si favoured its accumulation in bean plants with and without K deficiency, particularly when Si was supplied via SiRO compared to via SiLE (Fig. 2c).

**Figure 3:**
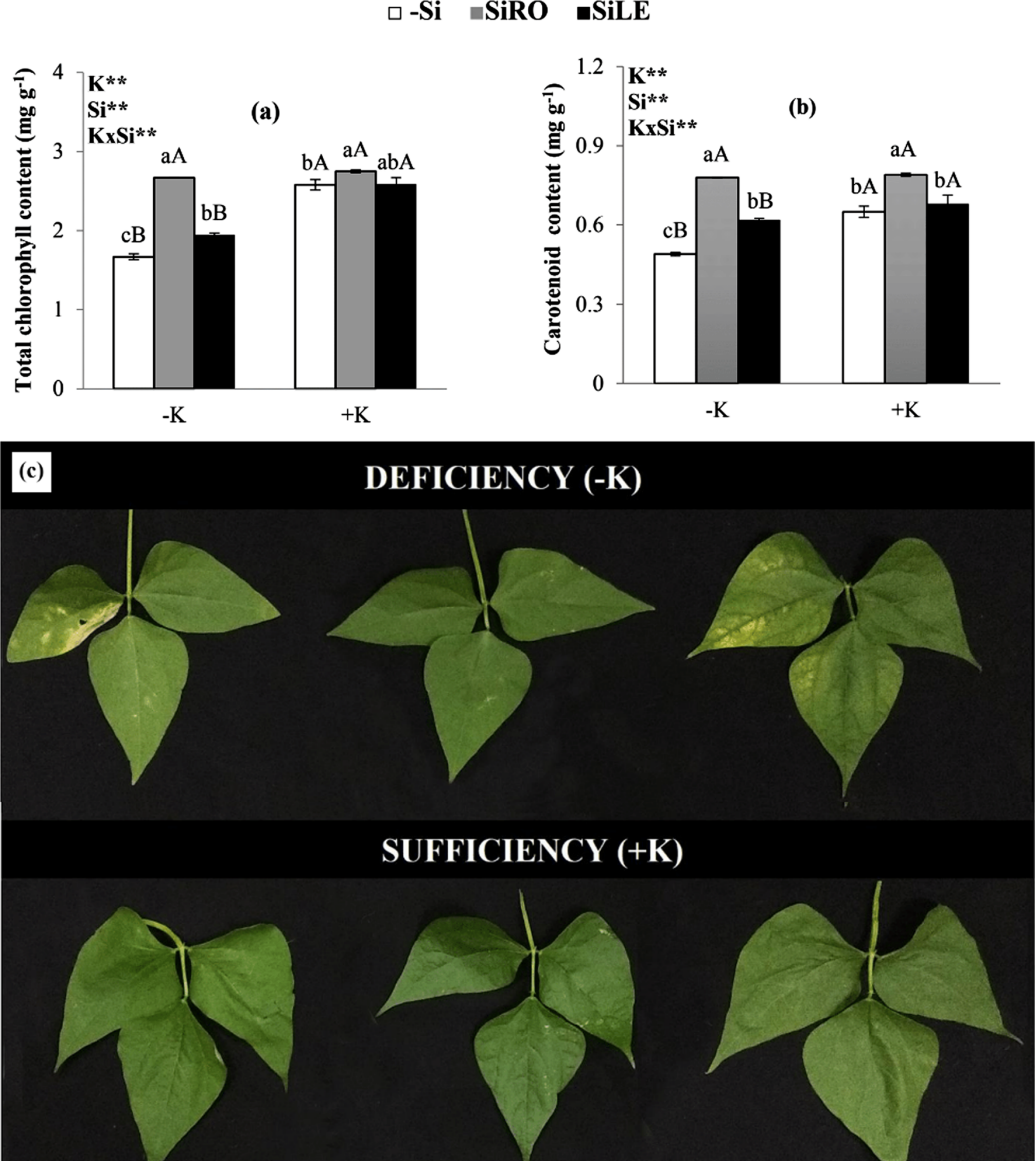
Total chlorophyll content (**a**), carotenoid content (**b**) and chlorosis in oldest leaves (**c**) of bean plants cultivated in a hydroponic system under deficiency (–K) and sufficiency (+K) of K, with Si supplied via nutrient solution (root) (SiRO), leaf spraying (SiLE), and control (–Si). The error bars in the figures represent standard error. Different letters, lower case between the supply of Si in the same concentration of K, and upper case between concentrations of K in the same form of supply of Si, indicate differences (P < 0.05, Tukey's test) between treatments.

Potassium deficiency in relation to its sufficiency decreased total content of chlorophyll and carotenoids in control plants (–Si) and those under Si foliar spray. Si (Fig. 3a,b). However, in K-deficient plants, SiRO treatment compared to the treatments SiLE and controls (–Si) resulted in higher total contents of chlorophyll and carotenoids (Fig. 3a,b). In plants under sufficient levels of K in the nutrient solution, only those treated with SiRO had increased the total contents of chlorophyll and carotenoids (Fig. 3a,b).

Bean plants cultivated in K-deficient nutrient solution exhibited chlorosis followed by necrosis on the edges of the oldest leaves. It was visually evident that this K deficiency symptom was alleviated by the supply of Si, especially provided via SiRO treatment (Fig. 3c).

**Figure 4:**
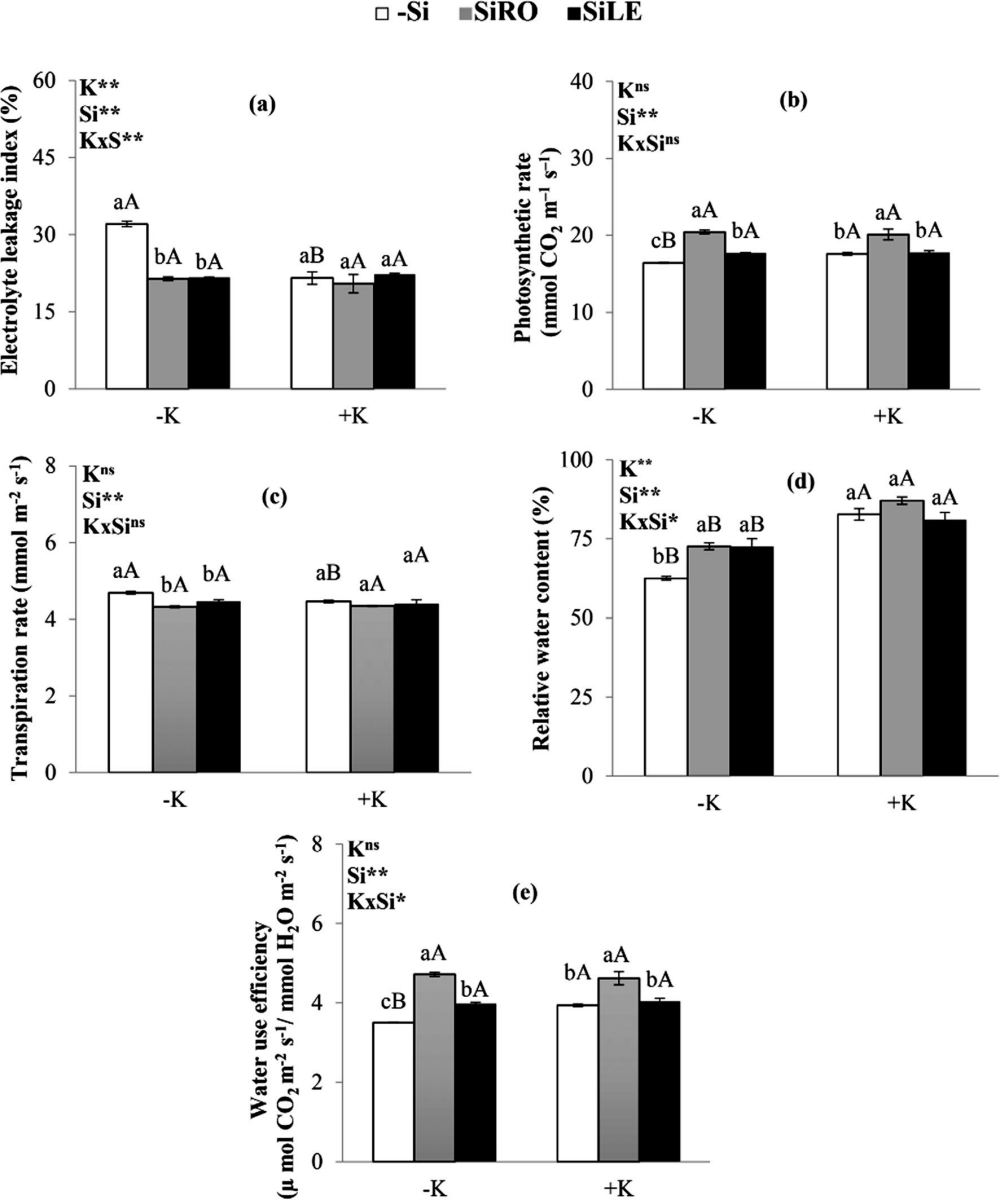
Electrolyte leakage index (**a**), photosynthetic rate (**b**), transpiration rate (**c**), relative water content (**d**) and water use efficiency (**e**) of bean plants cultivated in a hydroponic system under deficiency (–K) and sufficiency (+K) of K, with Si supplied via nutrient solution (root) (SiRO), leaf spraying (SiLE), and control (–Si). The error bars in the figures represent standard error. Different letters, lower case between the supply of Si in the same concentration of K, and upper case between concentrations of K in the same form of supply of Si, indicate differences (P < 0.05, Tukey's test) between treatments.

Plants stressed by K deficiency had increased electrolyte leakage (Fig. 4a), reduced photosynthesis rates (Fig. 4b), raised transpiration rates (Fig. 4c) and lowered relative water content (Fig. 4d) and water use efficiency (Fig. 4e) compared to plants with sufficient K levels. However, the Si supply, mainly with SiRO treatment, alleviated stress in K-deficient plants, as it increased photosynthesis, relative water content, and water use efficiency, in addition to minimizing transpiration rates and electrolyte leakage (Fig. 4a–e).

**Figure 5:**
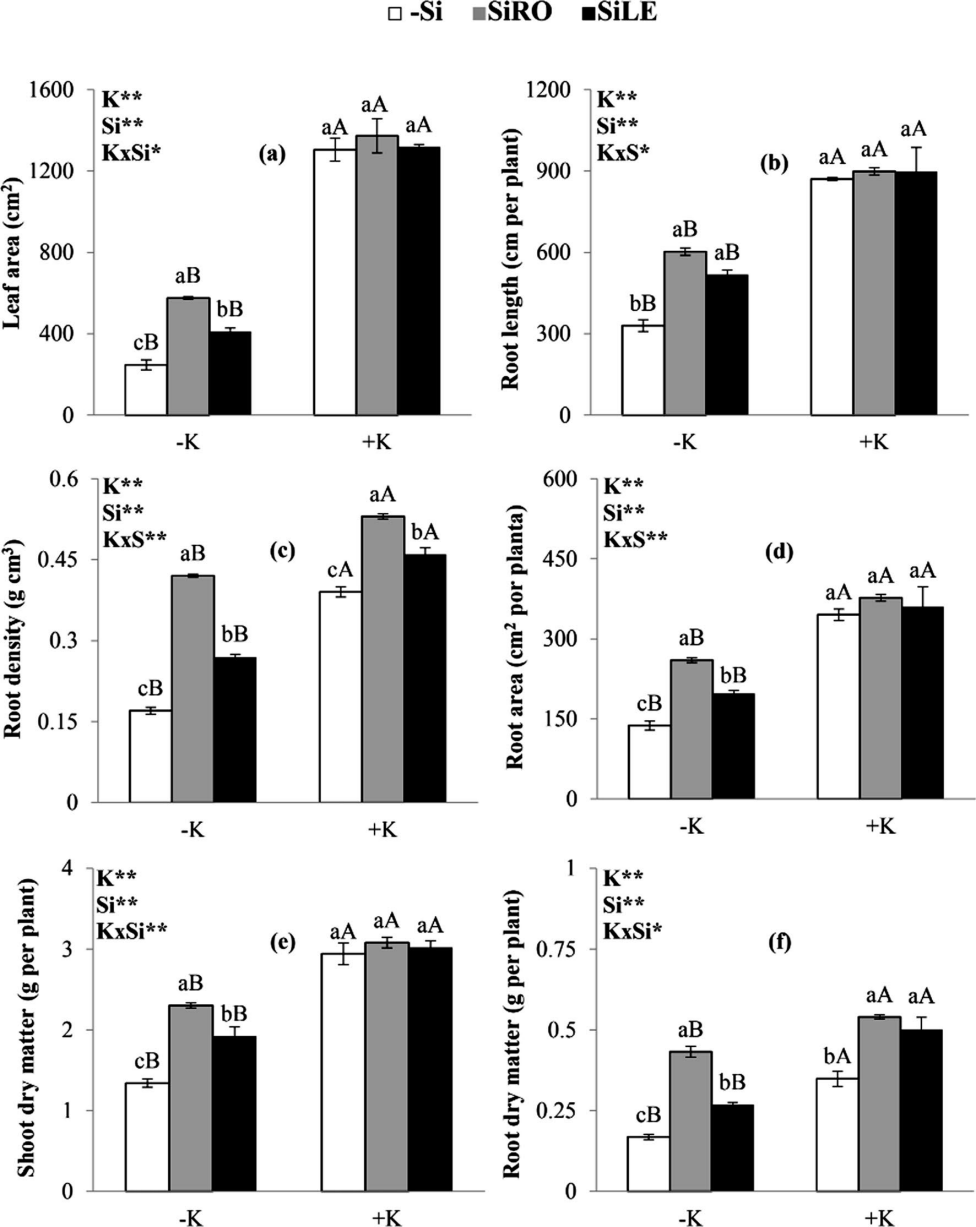
Leaf area (**a**), root length (**b**), root density (**c**), root area (**d**), shoot dry matter (**e**) and root dry matter (**f**) of bean plants cultivated in a hydroponic system under deficiency (–K) and sufficiency (+K) of K, with Si supplied via nutrient solution (root) (SiRO), leaf spraying (SiLE), and control (–Si). The error bars in the figures represent standard error. Different letters, lower case between the supply of Si in the same concentration of K, and upper case between concentrations of K in the same form of supply of Si, indicate differences (P < 0.05, Tukey's test) between treatments.

Bean plants cultivated in K-deficient solution had reduced the leaf area (Fig. 5a), root length (Fig. 5b), root density (Fig. 5c), and root area (Fig. 5d), in addition to shoot (Fig. 5e) and root dry matter (Fig. 5f).

**Figure 6:**
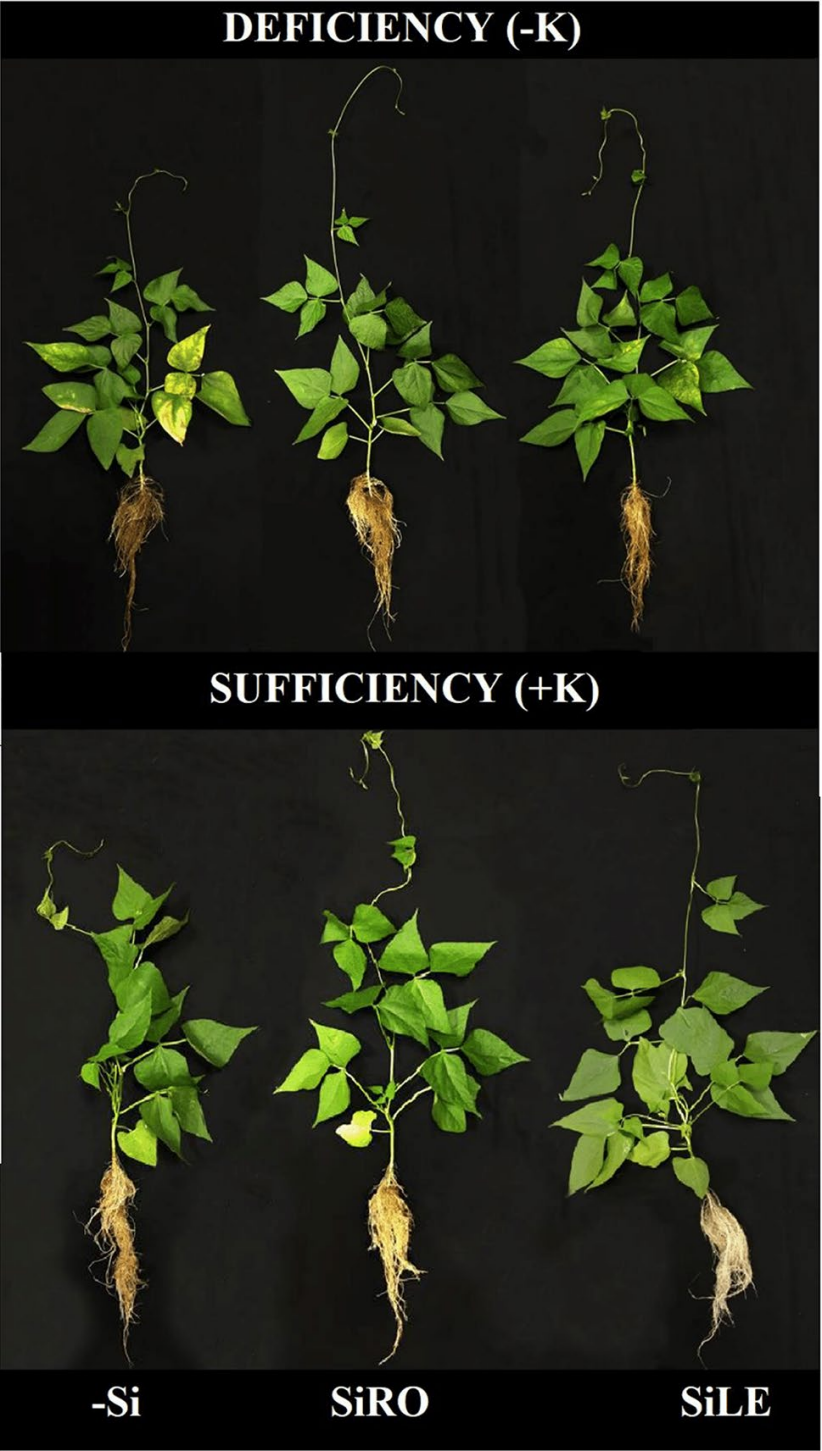
Si effect on shoot and root growth of bean plants cultivated in a hydroponic system under deficiency (–K) and sufficiency (+K) of K, with Si supplied via nutrient solution (root) (SiRO), leaf spraying (SiLE), and control (–Si).

However, K-deficient plants supplied with Si by both application methods had increased leaf area (Fig. 5a), root length (Fig. 5b), root density (Fig. 5c), and root area (Fig. 5d), as well as shoot (Fig. 5e) and root dry matter (Fig. 5f), highlighting root over leaf application. On the other hand, K-sufficient plants, SiRO or SiLE only increased root density and root dry matter. These results were also visualized from photographic records of the plants (Fig. 6).

## Discussion

Foliar application of silicon is effective in providing this beneficial element to plants. The emergence of new potential sources for foliar application requires studies that assess the efficacy of supplying Si to plants, as well as its effects on biological indicators^17,18^. It is known that increasing the concentration of Si in solution can increase foliar uptake if there is no high rate of polymerization. Si in solution, being in monomeric form (H_4_SiO_4_), is absorbed passively in legumes^19^. However, the increase in the concentration of this element in solution can induce the beginning of polymerization, forming polysilicic acid and subsequently, amorphous silica^20^. The Si in the plant predominates as amorphous silica and a small amount as monosilicic acid^21^. Thus, increasing the Si content in the plant is important to maximize its benefit, especially provided by foliar spray on plants that do not accumulate the beneficial element. Si foliar application was agronomically feasible due to the accumulation of this element in the bean plants, total chlorophyll content, QEPS II and consequent rise in dry matter production, irrespective of element source (Fig. 1). A similar result was found in bean plants by Barros et al.^22^ when Si was supplied via leaves at concentrations of 2 and 4 ml L^−1^. Furthermore, the two Si sources were efficient in increasing the element accumulation for the bean crop, as also found by Jafarei et al.^23^ who applied 3.6 g L^−1^ of Si by foliar via.

The accumulation of Si in plants prevented damage to pigments, being the first report of the effect of Si via foliar with these sources studied in bean plants grown in hydroponic system. This effect is due to Si deposition in the leaf epidermis, which protects the photosynthetic organelles^5^, and also due to the increased activity of antioxidant enzymes^24^. Furthermore, Si is involved in the formation of pigments^25^ and the protection of chloroplasts from aging^26^.

The SiNaK source performed better at increasing total chlorophyll content and the QEPS II when compared to SiK, although not sufficient to affect dry matter. This Si source stands out due to the sorbitol in its composition, which provides greater stability in the solution, reducing the polymerization process of the element^27^ on the leaf surface. The better performance of stabilized Si sources similar to the one used in our study compared to potassium silicate considered standard was also investigated by Souza Júnior et al.^16^, who obtained an increase in Si accumulation, chlorophyll content and quantum efficiency of photosystem II and consequently, an increase in the production of dry matter of cotton plants.

Bean plants cultivated in K-deficient nutrient solution (0.2 mmol L^−1^ of K) had lower accumulation of the nutrient when compared to plants with sufficient levels, which indicates nutritional stress (Fig. 2a). However, when these K-deficient plants were provided Si and especially in SiRO treatment, they had increase in accumulated K compared to controls (–Si). This may have occurred because Si stimulated H^+^-ATPase activity, enzymes directly linked to K uptake by plants^28^. This increased K accumulation in deficient plants was found in soybean supplied with Si via nutrient solution^8^. The Si supply to K-deficient plants, especially through the roots, enhanced K use efficiency by the bean plant compared to controls (Fig. 2b), due to the ability of Si to increase the element uptake (Fig. 2a) and the physiological processes associated with biomass production.Bean plants grown under potassium deficiency compared to K sufficiency indicated reduced Si accumulation only in SiRO treatment (Fig. 2c). As such, K deficiency induced less Si accumulation even in plants that received the beneficial element via nutrient solution, a finding also reported for barley plants^14^.

There was a greater accumulation of Si in common bean when the element was supplied via SiRO compared to SiLE in plants with or without K deficiency (Fig. 2c).

There was greater Si accumulation in bean plants when the element was supplied via SiRO compared to SiLE, in plants with or without K deficiency (Fig. 2c). This occurred since the Si supplied in the nutrient solution makes the element available throughout the crop cycle, whereas foliar application was carried out only in four stages.

The Si foliar application increased the accumulation of this element in relation to controls (–Si), in plants with and without K deficiency (Fig. 2c). This indicates that foliar spray increased Si uptake in the bean plants, a result also found in bean^22,29^ and okra plants^30^.

The K deficiency reduced total chlorophyll and carotenoid content in relation to sufficiency of the macronutrient only in controls (–Si) and in SiLE treatment (Fig. 3a,b). The cultivation of plants under low K-content in the nutrient solution decreased element uptake, causing decline in total chlorophyll content (Fig. 2a), a finding also reported for sorghum^5^. This is because the lack of this nutrient induces chlorophyll degradation as it induces oxidative stress, given the increase in reactive oxygen species and putrescine content, a compound that becomes toxic to plants when in high concentrations^1,5^.Thus, K deficiency resulted in chlorosis and necrosis on the edges of the oldest leaves (Fig. 3c), as previously reported by Prado^1^, Barker and Pilbeam^2^, Jones Jr.^3^ and Miranda et al.^4^.

In K-deficient plants, both methods for Si supply favoured an increase in total chlorophyll and carotenoid content compared to controls (–Si), especially in SiRO treatment (Fig. 3a,b), a difference that is clearly visible (Fig. 3c). The beneficial effect of Si supplied via nutrient solution in K-deficient plants on increasing chlorophyll content has also been reported for other species such as sorghum^5^ and barley^14^. Potassium deficiency in bean plants, with no addition of Si, increased electrolyte leakage in relation to plants with sufficient K contents (Fig. 4a). This occurred because K deficiency decreased intracellular pH, raising amine oxidase activity and stimulating the accumulation of reactive oxygen species, which oxidize compounds in the cell membrane^5^. However, both methods of Si application promoted less electrolyte leakage in K-deficient plants than in those that did not receive Si (–Si) (Fig. 4a). This finding is corroborated Miao et al. by^8^ in soybean plants that received Si via nutrient solution. This beneficial effect of Si in reducing electrolyte leakage is due to the element inducing greater plasma membrane protection^10^, possibly since it increased carotenoid content (Fig. 3b). Carotenoid is a non-enzymatic antioxidant that eliminates singlet oxygen (^1^O_2_), especially toxic oxygen reactive species, which leads to lipid peroxidation, resulting in a loss of cell electrolytes^31,32^ and stability of the lipid bilayer membrane^33^. Furthermore, the increase in carotenoids caused by Si treatment is important for the functioning of the photosynthetic apparatus, as it acts as an accessory in light uptake for photosynthesis and protects the chlorophyll from photooxidation in the reaction centre^34^.Plants stressed by K deficiency decreased their photosynthesis rates only in control plants (–Si) (Fig. 4b). This effect is due to the K deficiency decreasing the total chlorophyll content (Fig. 3a) and increasing electrolyte leakage (Fig. 4a), a finding also reported by other authors in sorghum plants^5,6^.

Beans plants cultivated under K-deficiency and receiving Si experienced an increase in the photosynthesis rate compared to that in controls (–Si), highlighting the application of SiRO (Fig. 4b), as observed in sorghum plants^5^. This effect is due to the SiRO treatment having increased K accumulation (Fig. 2a), as well as total chlorophyll (Fig. 3a) and carotenoid content (Fig. 3b).

This increase in pigments promoted by Si favoured the photosynthetic rate by increasing electron transport and activating genes (PetH; Os09g26810 and Os04g38410) related to photosynthesis^35^, and also by regulating the Rubisco enzyme^36,37^. Potassium deficiency without adding Si increased leaf transpiration rate (Fig. 4c) and decreased the relative water content of the control plant (Fig. 4d), since this nutrient regulates osmosis^1,6^. SiRO or SiLE treatment decreased foliar transpiration (Fig. 4c) and increased relative leaf water content (Fig. 4d) only in the K-deficient plants that did not receive Si (–Si). The beneficial effect of Si on the plant’s relative water content has been reported in K-deficient sorghum^6^. This effect is due to the formation of a silica gel layer that binds cellulose to epidermal cells, minimizing water loss^10^, as well as the increased activity of aquaporin, a protein associated with increased water transport in the plant^6^. K-deficiency also decreased water use efficiency in control plants (–Si) (Fig. 4e). This is because such deficiency reduces photosynthesis (Fig. 4b) and increases the leaf transpiration rate (Fig. 4c), resulting in low water use efficiency, a fact reported for other crops such as sorghum^6^ and cotton^38^. This damage caused by K deficiency in the efficient use of water is of concern given its importance in plant metabolism and its environmental consequences^1^. However, the water use efficiency in K-deficient plants increased with Si application in both methods and mainly in SiRO treatment (Fig. 4e). This beneficial effect of Si in raising water use efficiency is due to the increase in photosynthesis (Fig. 4b) and decrease in transpiration rate (Fig. 4c). The benefit of Si in increasing the efficiency of water use in a K-deficient plant is of great practical importance. This is because climate change has induced prolonged droughts in crops, restricting water availability, harming the physiological aspects of the crop^39^. In this sense, silicon could be an option to increase the efficiency of water use, minimizing damage to crops, especially in K deficient soils.

K-deficiency caused a decrease in plant growth (Figs. 5a–f and 6). This has been widely reported in the literature, given the functions of K in plants^1–3^, whose deficiency compromises biological variables, as previously mentioned.

On the other hand, both methods of Si application increased plant growth variables, especially via SiRO (Fig. 5a–f). A similar result of increased dry matter in K-deficient plants submitted to Si application via nutrient solution was obtained in other species such as soybean^8^ and sorghum^5,6^.

The benefits of Si to attenuate K deficiency in bean plants can be explained by nutritional and physiological improvements. This benefit is because Si increased the accumulation of K in the bean plant (Fig. 2a). Furthermore, another benefit is the induction of antioxidant defence mechanisms in the plant. This is because the Si uptake increased the total chlorophyll (Fig. 3a) and the content of antioxidant carotenoid compounds (Fig. 3b) and, decreased the leakage of electrolytes (Fig. 4a); consequently, favouring the photosynthesis rate (Fig. 4b).

Si also maintained a favourable water status in the plant, given the decrease in transpiration rate (Fig. 4c), which promoted an increase in water content (Fig. 4d) and, in turn, more efficient use (Fig. 4e). Thus, improving physiology and nutrition by supplying Si to the K deficient plant increased the efficiency of using the macronutrient to convert it into biomass (Fig. 2b), hence improving plant growth. The beneficial effects of root-applied Si on the physiology and growth of K-deficient plants have also been reported in species other than common bean^5,6,8^. Importantly, SiLE improved the growth variables of K-deficient bean plants that did not receive Si (–Si). This may be due to the effect of foliar Si in increasing total chlorophyll and carotenoid content as well as photosynthesis and water use efficiency, in relation to controls (–Si). The benefits of leaf-applied Si in alleviating K deficiency in bean plants have not been reported in the literature, as existing studies supplied the element only via nutrient solution. The present study demonstrates the mitigating effect of Si on K deficiency, especially supplied via nutrient solution, but foliar application is a viable alternative in bean plants. This result makes it clear that Si root uptake by bean plants was effective, being sufficient to reach the shoot and mitigate K deficiency. This indicates that although the bean plant is classified as non-Si-accumulating^19^, it uptakes the element but less than a Si-accumulating plant. Therefore, it is worth advancing research to better understand the processes and identify Si carriers in this important species. Finally, SiRO or SiLE treatments had little effect on the growth of K-sufficient plants, since only root density and dry matter increased. As such, the present study showed that the most important role of Si is when plants are under nutritional stress compared to those under sufficient levels, a fact reported by other authors^40,41^, as this element is not essential for plants.

This research elucidated how Si acts to overcome K deficiency in common bean, which may have global implications, as according to Choudhary et al.^42^, the crop is cultivated in many areas of America, Europe, Africa and Asia.

However, further research on species of the same family as the bean and under field conditions is encouraged to calibrate the doses of Si, as well as areas with Si- and K-deficient soils should be prioritized.

## Material and methods

### Local and growing conditions

Two experiments were conducted in a hydroponic growing system in the greenhouse at the School of Agricultural and Veterinarian Sciences (UNESP), Jaboticabal, Brazil.

Seeds of common beans (cv. BRS Estilo) were obtained from the Brazilian Agricultural Research Corporation of the Ministry of Agriculture, Livestock and Food Supply, Brazil.

The use of plant parts in the present study complies with international, national, and/or institutional guidelines. This research was not conducted with endangered species and was conducted in accordance with the is in accordance with the Declaration of IUCN Policy on Research Involving Endangered Species.

The first experiment aimed to obtain the best Si concentration and source for Si foliar spray, which lasted 115 DAE (days after emergence). Based on results of the first experiment, a second experiment was conducted to evaluate the effect of Si on the physiology and dry matter yield of K-deficient bean plants, being maintained until the emergence of K deficiency symptoms, corresponding to the phenological stage R5 (28 DAE). The relative air humidity and maximum and minimum air temperature were recorded throughout the experimental period. There was a high variation in the average relative humidity (34.3 ± 9%|32.8 ± 8%), minimum temperature (17.9 ± 7 °C|19.5 ± 5 °C) and maximum temperature (44.8 ± 8 °C|38.6 ± 7 °C) to the first and second experiment respectively. High temperatures may have induced plants to possible stresses, considering that the average temperature for optimal beans crop growth is between 18 and 24 °C^43^.

### Growing conditions

For the first experiment, the seeds were sown in trays. Then the seedlings at five DAE were transplanted to 7 dm^3^ polypropylene pots (upper diameter: 16 cm; lower diameter: 11 cm; height: 33 cm), filled with 6 dm^3^ medium texture sand, previously washed with water, 1% HCl solution and deionized water, maintaining two plants per pots. These pots were irrigated daily with nutritive solution to maintain 70% water retention capacity in the substrate.

For the second experiment, the seeds were also sown in trays, and the seedlings at five DAE were transplanted to polypropylene pots (length: 44 cm; width: 19 cm and height: 14 cm, with capacity for 10 litters), also filled with the nutritive solution.

The nutrient solution used in both experiments was proposed by Hoagland and Arnon^44^. The nutrient solution concentration during the first and second week of growth was maintained at 10 and 25%, respectively, as indicated by the authors^44^. From the third week until the end of the experiments, the concentration was raised to 50%. The pH value of nutritive solution was maintained between 5.5 and 6.5, corrected using NaOH (1 mmol L^−1^) and HCl (1 mmol L^−1^) solution. In the second experiment, the hydroponic solution was modified with different levels of K, as per the treatment (Table 1), and renewed every week to replenish the water, Si and nutrients absorbed by the plants.

**Table 1:** Amount of K provided and adjustment between control (–Si), Si via roots (SiRO) and Si via leaf spraying (SiLE) treatments in the second experiment.

K supply route	−K			+K		
	-Si	SiRO	SiLE	-Si	SiRO	SiLE
	mmol L^−1^					
Via root	0.2	*	0.2	6	5.8*	6
Via leaf	0.5	0.5	**	0.5	0.5	**
Total K	0.7	0.7	0.7	6.5	6.5	6.5

*Received 0.2 mmol K via SiNaK; ** received 0.5 mmol via SiNaK.

### Experimental design

The first experiment was carried out in a complete randomized block design in a 2 × 4 factorial scheme, with two sources of Si: sodium and potassium silicate stabilized with sorbitol (SiNaK) (113.4 g L^−1^ of Si and 18.9 g L^−1^ of K_2_O, pH 11.8) and potassium silicate without stabilizer (SiK) (128 g L^−1^ of Si and 126 g L^−1^ of K_2_O, pH 12.0). Four concentrations: 0.0; 5.4; 10.8 and 16.2 mmol L^−1^ of Si. All treatments were conducted in four replicates.

The second experiment was arranged in completely randomized blocks in a 2 × 3 factorial scheme, with two concentrations of K in the nutrient solution: deficient (−K) (0.2 mmol L^−1^ of K) and sufficient (+K) (6 mmol L^−1^ of K), and two methods of Si supply: in nutrient solution via roots (SiRO) (2 mmol L^−1^ of Si), foliar (SiLE) (5.4 mmol L^−1^ of Si per application), in addition to the control (–Si) (0 mmol L^−1^ of Si), in four repetitions.

### Si application and K adjustment

For the first experiment, Si foliar applications (SiNaK and SiK) were performed at three stages of development: V4 (emergence of the 3rd trifoliate leaf), R6 (flowering – opening of the first flower) and R7 (pod formation). The volume of solution applied varied according to plant size and 8, 16 and 24 ml of the solution were sprayed at stages V4, R6 and R7 respectively.

For the second experiment, SiNaK was applied as a Si source. In the SiRO treatment, the Si supply via root was performed in a nutrient solution throughout the experiment.

For foliar application in the second experiment (SiLE treatment), a solution with a concentration of 5.4 mmol L^−1^ of Si (SiNaK) was made and applied to the leaves manually. The volume of Si solution applied increased according to plant size, with 0.56; 0.84; 1.12 and 1.40 ml of the silicate solution per plant for the first, second, third, and fourth sprays, respectively, at 8, 13, 18 and 23 DAE.

The solutions used for foliar spray in both experiments were adjusted with a solution of NaOH and HCl to maintain a pH of 6.0 ± 0.2. Silicon was applied to the leaves immediately after solution preparation.

The SiNaK and SiK sources contains K in its composition, after Si sprayings, foliar applications were performed with potassium chloride to K balance in the treatments. In the second experiment, the K provided by the SiNaK source was also adjusted for the root supply (Table 1).

It is important to highlight that 0.7 mmol L^−1^ of K from SiNaK does not meet the demand of 6 mmol L^−1^ suggested by Hoagland and Arnon ^44^ to supply K to plants, and nutrient deficiency of this nutrient is expected.

Temperature (^o^C) and relative humidity (%) in both experiments were measured during foliar applications, obtaining values between 9 and 22 °C and 60 and 80% respectively.

### Plant analysis

In the first experiment, assessments were conducted in stage R7, and at twenty-five DAE for the second experiment, both in the upper third of the trifoliate leaf.

#### Quantum efficiency of photosystem II and Gas exchange parameters

In the first experiment, the quantum efficiency of photosystem II (QEPII) was measured with a fluorimeter (Opti-Science®-Os30P +).

In the second experiment, Gas exchange parameters were determined between 9–11 am, using four replicates for each treatment. Photosynthetic rate and transpiration rate were measured using an open infrared gas analyser (IRGA LcPro-SD, ADC BioScientific Ltd., Hoddesdon, Reino Unido). The IRGA chamber was irradiated with a photosynthetic photon flux density of 1200 μmol m^−2^ s^−1^ and under ambient CO_2_ concentration (400 ± 10 μmol m^−2^ s^−1^). Water use efficiency (WUE) was calculated as net photosynthetic rate (A) per transpiration rate (E): WUE = A/E.

#### Total chlorophyll and carotenoid content

Total chlorophyll (a + b) and carotenoid content in the first and second experiments were measured by an absorbance spectrophotometer at 663 nm for chlorophyll a, 647 nm for chlorophyll b, and 470 nm for carotenoids. Pigment concentrations were determined according to Lichtenthaler and Wellburn^45^.

#### Electrolyte leakage and relative water content

In the second experiment, the electrolyte leakage index and relative water content (RWC) were measured according to the methodology proposed by Dionisio-Sese and Tobita^46^ and González and González-Vilar^47^, respectively.

#### Plant growth analysis and dry matter

Leaf area of the plant was measured with a LI – 3100 Area Meter®. Moreover, the root system was analysed using the Delta-TScan system and the length measured using the method developed by Harris and Campbell^48^. Root density was calculated by the ratio between root length and solution volume in the pot.

The plants were cut and separated into shoots and roots. Next, the samples were washed with deionized water, 0.1% neutral detergent solution, 0.3% HCl solution and again with deionized water, and dried in a forced air oven at a temperature of 65 °C ± 5, until reaching constant weight. After drying the samples, root and shoot dry matter were obtained, followed by grinding in a Wiley mill.

#### Si accumulation and K use efficiency

To determine the Si content, shoot dry matter (first experiment) and shoot and root dry matter (second experiment) were used. For Si analysis, the samples were extracted following the methodology proposed by Kraska and Breitenbeck^49^, and measured in a spectrophotometer at 410 nn to obtain Si content, following the methodology described by Korndörfer et al.^50^.

In the second experiment, K content was analysed by digestion in nitric perchloric acid solution, followed by atomic uptake spectrophotometer reading according to the methodology described by Zasoski and Burau^51^. Based on values of Si, K and dry matter, the accumulation of these elements in the entire plant (shoots and roots) was calculated following the formula: Element accumulation = ((element content g kg^−1^) * (plant mass g per plant))/1000.

K use efficiency was estimated considering the dry matter production and K content, according to the methodology described by Siddiqi and Glass^52^: (entire dry matter production)^2^/(accumulation of nutrient in the entire plant).

### Statistical analysis

Experimental data were submitted to analysis of variance applying the F-test, and when significant for qualitative variables, to Tukey’s test (p < 0.05) to compare the means, using SAS statistical software 9.2^53^. The data were checked for outliers (Dixon’s Q test), normality (Shapiro–Wilk test) and homogeneity of variances (Levene’s test).

## Conclusions

Si foliar spray was agronomically feasible for bean plants, particularly the solution containing silicate of sodium and potassium stabilized at a concentration of 5.4 mmol L^−1^.

Si supplied via nutrient solution or foliar application mitigated K-deficiency stress in the bean plant due to improvements in nutritional, physiological and growth variables. We emphasize the Si supply via nutrient solution compared to foliar application, although the latter also exhibited attenuating properties.
